# Lactate for Predicting the Prognosis of Multi-Drug Poisoned Patients

**DOI:** 10.4314/ejhs.v33i1.14

**Published:** 2023-01

**Authors:** Durdu Mehmet Uzucek, Dervis Yildiz, Ahmet Burak Urfalioglu, Satuk BugraYapici, Kemal Sener, Akkan Avci, Sadiye Yolcu

**Affiliations:** 1 Department of Anaesthesiology, Chettinad Hospital and Research Institute, Tamil Nadu, India

**Keywords:** Emergency, multi-drug, poisoning, prognosis, lactate

## Abstract

**Background:**

We aimed to compare serum lactate levels of multi-drug poisoned patients to determine whether knowing the level may help emergency clinicians in predicting the patients' prognoses.

**Methods:**

The patients were divided into two groups according to the number of kinds of drugs taken (Group 1: patients took 2 kinds of drugs; Group 2: patients took 3 or more kinds of drugs). The groups' initial venous lactate levels, lactate levels before discharge, lengths of stay in the emergency department, hospitalisation units, clinics, and outcomes were recorded on the study form. These findings of the patient groups were then compared.

**Results:**

When we evaluated the first lactate levels and lengths of stay in the emergency department, we found that 72% of the patients with initial lactate levels ≥13.5 mg/dL stayed more than 12 hours in the emergency department. Twenty-five (30.86%) patients in the second group stayed ≥12 hours in the emergency department, and their mean initial serum lactate level was significantly related (p=0.02, AUC=071). The mean initial serum lactate levels of both groups were positively related with their lengths of stay in the emergency department. The mean initial lactate levels of patients who stayed ≥12 hours and those who stayed <12 hours in the second group were statistically significant, and the mean lactate level of the patients who stayed ≥12 hours in the second group was lower.

**Conclusions:**

Serum lactate levels may be helpful in determining a patient's length of stay in the emergency department in the case of multi-drug poisoning.

## Introduction

Suicidal drug intake is one of the most important clinical situations for emergency departments all over the world, and it is much more common in the young population. Asymptomatic patients may be discharged from the emergency department after 6 hours of observation. These patients should undergo gastric decontamination via charcoal administration if they present within 1 hour of the drug intake. A complete blood count, electrolytes, urine analysis, arterial blood gases, blood urea, liver enzyme tests, and ECG should be evaluated according to the type of the drug (1).

Delayed toxicity occurs with extended-release medical drugs, so patients who have taken these drugs should be followed for 24 hours at a minimum (2). Similarly, multi-drug poisonings are much more confusing, and discharge decisions can become much more complicated in these patients. Serum lactate levels are used to determine the severity and the progression of many diseases. Levels higher than 4 mmol/L indicate and correlate with worse prognoses (3). Shock, sepsis, cardiopulmonary arrest, trauma, epilepsy, and toxins may cause higher serum lactate levels. In addition, high lactate levels are related to higher mortality rates. Decreased lactate clearance is associated with high mortality rates in patients with sepsis, trauma, burns, and other acute diseases. Clinicians should consider multifactorial causes in patients with high serum lactate levels (4).

The discharge decision for a patient who took a single drug is easier. The specific treatment and pathway are known. In contrast, multi-drug poisoning requires much more effort, and the treatment is complicated. Each drug should be treated and followed according to its pharmacology.

In this study, we aimed to compare serum lactate levels of multi-drug poisoned patients (2 drugs/3 or more drugs) to determine whether knowing the level may help emergency clinicians in predicting the patients' prognoses, lengths of stay in the emergency department, and discharge decisions.

## Materials and Methods

**Study design**: In this retrospective study, after receiving ethical approval, we included multidrug poisoned patients who presented to our emergency department between 1 January 2017 and 1 October 2019. The ethics committee of the Adana City Training and Research Hospital approved the study (Date: 06/11/2019, meeting number: 43, decision number: 600).

**Selection of participants**: We included patients who took two or more types of drugs. The patients' data were obtained from the hospital automation system. The patients were divided into two groups according to the number of kinds of medication they reportedly ingested (Group 1: patients took 2 kinds of drugs; Group 2: patients took 3 or more kinds of drugs). The groups' demographic data, initial venous lactate levels, lactate levels before discharge, lengths of stay in the emergency department, hospitalisation units, hospitalisation clinics (inpatient clinic/ICU), and outcomes were recorded on the study form. These findings of the patient groups were then compared. Venous blood gas analyses were performed with an AKINLAB Radiometer ABL800 (İstanbul, Turkey), and the lactate levels were obtained from the venous blood gas analyses.

Patients under the age of 18, those with chronic multiple drug use due to co-morbidities, those with non-drug intoxication, pregnant women, those who were referred to other hospitals for intensive care (ICU) beds, patients with missing data and single drug poisoning were determined as the exclusion criteria.

**Statistical analyses**: Statistical comparisons were performed using the statistical software package SPSS 25.0 (SPSS Inc., Chicago, IL, USA). The Shapiro-Wilk test was used to check for a normal distribution. The normally distributed variables were evaluated with independent sample t tests. The non-normally distributed variables were analysed using the Mann-Whitney U test. The categorical variables were expressed in frequencies and percentages. Chi-square tests were used to compare the categorical data. Definitive statistics were expressed as the mean ± SD and median (interquartile range, IQR). A p value <0.05 was considered significant.

**Ethical Approval:** The Ethics Committee of the Adana City Training and Research Hospital approved the study (Date: 06/11/2019, meeting number: 43, decision number: 600). This manuscript was carried out in accordance with the Declaration of Helsinki and Good Clinical Practice guidelines.

## Results

We included 124 patients (37 men, 29.8%; 87 women, 70.2%) in our study. The numbers of patients in group 1 (2 drugs) and group 2 (three or more drugs) were 43 (34.7%) and 81 (65.3%), respectively. Group 1 comprised 10 (23.3%) men and 33 (76.7%) women, and group 2 comprised 27 (33.3%) men and 54 (66.7%) women. We found no statistically significant difference between the groups according to gender (p=0.304) (Fischer's-chi square) (Table 1). None of the patients in group 1 underwent haemodialysis, but 1 (1.2%) patient from group two underwent haemodialysis. The difference between groups according to haemodialysis was not significantly different (p=1) (Fischer's chi-square).

**Table 1:** The study groups' demographics

Variable	Group 1 n (%) ± SD	Group 2 n (%) ± SD	*p
	43 (34.7%)	81 (65.3%)	
Sex (male/female)	10/33 (23.3%/76.7%)	27/54 (33.3%/66.7%)	0.304
Age (years)	29.8 ± 12.07	31.4 ± 12.31	0.497
Time to arrival at emergency department (hours)	2.03 ± 2.06	2.08 ± 2.09	0.908

* Fischer's chi-square

The mean age of group 1 was 29.8±12.07, and for group 2, this value was 31.4±12.31 (p=0.497). The mean arrival times (in hours) after drug intake were 2.03±2.06 for group one and 2.08±2.09 for group 2 (p=0.908). The first and last pH levels, first and last lactate levels, and lengths of stay in the emergency department and a comparison of the groups are given in Table 2.

**Table 2:** The first and last pH levels, first and last lactate levels, and lengths of stay in the emergency department and a comparison of the groups

	Group	Mean	*p
First pH	1	7.3881±0.04929	0.679
	2	7.3922±0.05371	
Last pH	1	7.3744±0.03432	0.216
	2	7.3833±0.04399	
First lactate (mg/dL)	1	17.5116±13.74144	0.929
	2	17.8025±18.89472	
Last lactate (mg/dL)	1	10.3488±5.76064	0.875
	2	10.6790±13.03057	
Length of stay in the emergency department (hours)	1	14.3953±20.51613	0.618
	2	12.8025±14.60259	

* Mann-Whitney U test

The mean first lactate levels and lengths of stay in the emergency department of the patients followed-up in the emergency department and discharged were positively correlated (p=0.01, r=0.232) (Pearson correlation). The same correlation was determined in group 1 (p=0.012, r=0.378). However, we found no significant correlation between the first lactate levels and lengths of stay in the emergency department in group 2 (p=0.125, r=0.172).

The mean of our study groups' lengths of stay in the emergency department was 13.35±16.82 (min: 3.00, max: 96.00) hours. Thirty-one (25%) patients stayed ≤12 hours in the emergency department, and 91 (75%) patients stayed >12 hours. This difference was significant between the groups.

**Figure 1:**
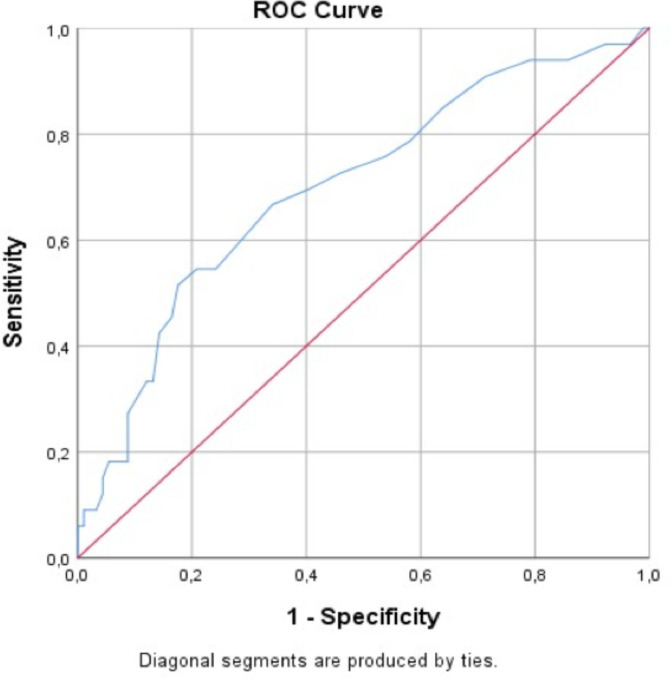
ROC curve for initial serum lactate levels and lengths of stay in the emergency department.

When we evaluated the first lactate levels and lengths of stay in the emergency department, we found that 72% of the patients with initial lactate levels ≥13.5 mg/dL stayed more than 12 hours in the emergency department (p=0.001, AUC=0.699) (Figure 1).

The sensitivity and specificity of patients in the second group who stayed ≥12 hours in the emergency department were determined according to their initial serum lactate levels. The serum lactate levels increased in 22 (17.7%) patients, did not change in 10 (8.06%) patients, and decreased in 92 (74.24%) patients. Eight (18.60%) patients in the first group and 35 (43.20%) in the second group stayed ≥12 hours in the emergency department.

**Figure 2:**
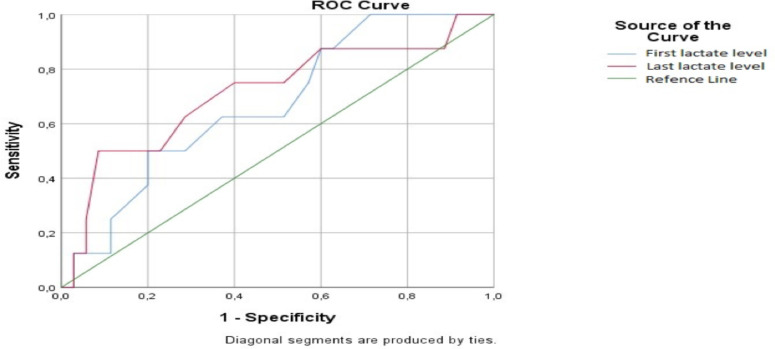
ROC curve for sensitivity and specificity in the last lactate levels of the first group at the 12-hour follow-up.

The patients whose last lactate levels were ≥10.5 mg/dL stayed ≥12 hours in the emergency department (sensitivity=62%, specificity =71%) (Figure 2).

**Figure 3:**
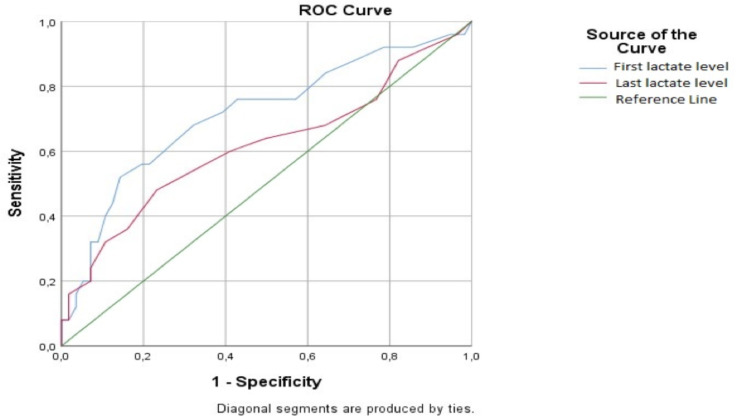
ROC curve for the mean initial and the last serum lactate levels of the patients staying ≥12 hours in the emergency department

Twenty-five (30.86%) patients in the second group stayed ≥12 hours in the emergency department, and their mean initial serum lactate level was significantly related (p=0.02, AUC=071). However, their last lactate levels were not related (p=0.098, AUC=0.61) (Figure 3).

Those with initial lactate levels ≥14.50 mg/dL stayed ≥12 hours in the emergency department (sensitivity=72%, specificity=60%). These measurements were not valuable for the last lactate levels of this group.

The mean initial serum lactate levels of both groups were positively related to their lengths of stay in the emergency department (p=0.01, r=0.232). The same correlation was determined for the first group (p=0.012, r=0.378), but the second group did not show a correlation (p=0.125, r=0.172).

The first group was evaluated according to their stay lengths and initial lactate levels. The mean initial lactate level of patients who stayed ≥12 hours (and those who stayed <12 hours) was not significantly different (p=0.194). This comparison in the second group was statistically significant, and the mean lactate level of the patients in the second group who stayed ≥12 hours was lower.

## Discussion

The management of multi-drug poisoning is much more difficult than that of single-drug toxicity. The algorithms are known for each pharmacologic agent, and the follow-up of these patients is clear. However, multi-drug poisoning presents much more frequently than single-drug poisoning (5), but lower ratios have been reported in the literature. Lionte et al. reported that more than 30% of the poisoned patients took multiple drugs (6).

In our study, most of the patients were female (70.2%) and young, as expected (7). Controversially, Brillo-Putze evaluated 419 intoxication cases and found that the number of male patients was higher than that of female patients (8). Similarly, as reported in the literature, most poisoning cases are young patients, and the mean age of our study group was about 30 years old (8,9).

A high serum lactate level occurs with tissue hypoxia and is known as a rapid and useful predictor for emergency clinicians. High initial serum lactate levels are related to worse prognoses and high ratios of ICU admission and mortality. Recently, our knowledge about lactate levels in sepsis, trauma, and poisoning patients have helped us predict patients' prognoses and management strategies (10–15).

In a large geriatric case series (1,987 patients), checking the serum lactate level was suggested as an alternative to taking routine vital signs, and high levels were associated with high risks of mortality (11).

In our study, the mean initial serum lactate levels were 17.51 mg/dL for group 1 and 17.80 mg/dL for group 2, and we found higher serum lactate levels in hospitalised patients than in discharged patients. Serum lactate levels were also associated with hospitalisation lengths. Cheug et al. reported that the mean initial serum lactate level of the patients in their study was 41.6 mg/dL, and this value was 106.2 mg/dL for the patients who died because of drug poisoning. Thus, the serum lactate level can be used as a good predictor of drug poisoning (12). In a single-drug poisoning study, Megarbane et al. reported that serum lactate levels cannot predict mortality in beta blocker poisonings (15), but most studies have been based on multi-drug poisonings, so additional data about single-drug poisoning are required.

The serum lactate level is also correlated with the COHb level in carbon single oxide poisoning. In addition, the serum lactate level can be used as a predictor of hospitalisation requirements and risk stratification (14).

Considering that multi-drug poisoning management is confusing for clinicians, a post-mortem study reported that toxin levels were lower in multi-drug poisoning patients (16). This is because the patient takes a lot of pills if it is a single-drug overdose but probably does not take as many pills in a multi-drug poisoning.

We compared the lactate levels of two groups of patients (2 kinds of drugs/3 or more kinds of drugs). According to our knowledge, this is the first study to evaluate serum lactate levels in multi-drug poisonings compared according to the number of kinds of pills. The first and last mean lactate levels were not significantly different between our study groups. The first lactate levels were related with lengths of stay in the emergency department in group one, but they were not related in group two. Similarly, this situation can be explained as above. The number of pills per drug decreases when the number of kinds increases.

We also determined that the patients with initial serum lactate levels ≥13.5 mg/dL stayed more than 12 hours in the emergency department. Given the crowdedness of emergency departments, the initial lactate levels of multi-drug poisoned patients can serve as a guide for bed and area planning.

In our clinic, we mostly follow up poisoning patients in our emergency department (92% for multi-drug poisoning), but this hospitalisation rate varies in a wide range (5.1%–64%) (17,18). A high hospitalisation rate may be related to the level of the emergency department. Similar to our study, Guven et al. reported a lower (7.8%) ICU hospitalisation rate (19).

In our study, we determined that the mean initial serum lactate levels of both groups were positively related with their lengths of stay in the emergency department. The same correlation was determined for the first group.

The first group was evaluated according to their stay lengths and initial lactate levels. The mean initial lactate levels of patients who stayed ≥12 hours and those who stayed <12 hours were not significantly different. This comparison in the second group was statistically significant, and the mean lactate level of the patients in the second group who stayed ≥12 hours was lower. When the number of the kinds of drugs increases, knowing whether the patient's length of stay in the emergency department was ≥12 hours and measuring a lower lactate level may be helpful in discharge.

In conclusion, our study is the first lactate research comparing poisonings from 2 drugs and ≥3 drugs. We found that initial serum lactate levels are related to the patient's length of stay in the emergency department. Serial lactate measurement may be helpful in making a decision on the patient's length of observation in the emergency department and discharge. When the crowdedness of emergency departments is considered, easily reachable markers such as the venous blood lactate level present a new frontier for planning and management. Multi-centre, comprehensive serial lactate measurements in multi-drug poisoning studies are required.

We could not evaluate the relationship between the lactate level and mortality as none of the patients in our study groups died. This was the limitation of this study.
